# Inconclusive flow cytometric surface light chain results; can cytoplasmic light chains, Bcl-2 expression and PCR clonality analysis improve accuracy of cytological diagnoses in B-cell lymphomas?

**DOI:** 10.1186/s13000-015-0427-5

**Published:** 2015-10-20

**Authors:** Andreja Brozic, Ziva Pohar Marinsek, Srdjan Novakovic, Veronika Kloboves Prevodnik

**Affiliations:** Department of Experimental Oncology, Institute of Oncology, Ljubljana, Slovenia; Department of Cytopathology, Institute of Oncology, Ljubljana, Slovenia; Department of Molecular Diagnostic, Institute of Oncology, Ljubljana, Slovenia

**Keywords:** B-cell lymphoma, Flow cytometry, Immunoglobulin light chains, Bcl-2, PCR clonality analysis

## Abstract

**Background:**

Flow cytometric immunophenotyping (FCI), is widely used in cytology for distinguishing between B-cell lymphoma (BCL) and reactive lymphocytic proliferations (RLP), mainly by identifying monotypic B-cell populations. Since this cannot always be determined by ratios of surface immunoglobulin light chains (sIg LCs) we wanted to assess if cytoplasmic immunoglobulin (cIg) LCs, Bcl-2 and polymerase chain reaction (PCR) based clonality analysis can improve accuracy of cytological diagnoses of BCL.

**Methods:**

Our study included 98 fine needle aspiration biopsies from lymph nodes suspicious for BCL with inconclusive sIg LCs. In all cases PCR clonality analysis was performed in order to determine immunoglobulin heavy chain (IGH) gene and T-cell receptor (TRC) gene rearrangement. In selected cases expression of Bcl-2 and cIg LC were determined by FC.

**Results:**

Thirty patients had lymphoma and 68 had reactive lymphocytic proliferations. Three patterns of sIg LCs staining were found: negative, dual positive and difficult to interpret. Percentage of lymphomas was highest in the dual positive group (75 %). Morphology coupled with cIg LCs determination and/or Bcl-2 expression was able to give a correct diagnosis in 83 % of cases. Molecular tests would have been misleading in 15 % of cases because 7/30 BCL were polyclonal and 8/68 RLP were monoclonal.

**Conclusions:**

Determination of cIg LCs, Bcl-2 expression and PCR clonality analysis of B cells improved accuracy of cytological diagnoses in BCL with inconclusive sIg LC. However, clonality determined by PCR was misleading in some cases.

## Background

Flow cytometric immunophenotyping (FCI), is widely used in cytology for distinguishing between B-cell lymphoma (BCL) and reactive lymphocytic proliferations (RLP). A few lymphoma types can be identified on the basis of an aberrant immunophenotype. However, differentiation between BCL and RLP is accomplished mainly by identifying monotypic populations of B cells [1]. This can be done reliably by determination of surface immunoglobulin (sIg) light-chains [2], using mostly the ratio between surface kappa (κ) and lambda (λ) light chains (LCs) [3–11].

However, in some cases, monotypicity cannot be determined by surface κ/λ ratio, because κ and λ are not expressed on B cells (negative sIg LCs) or both κ and λ LC can be detected in the same B cell population (dual positive sIg LCs). We found few reports mentioning cases with negative sIg LCs [12–17] and a few articles reporting cases with dual sIg LCs [17–23]. Some authors claim that absence of sIg LC is suggestive for B-cell lymphoma [12–14]. However, even normal B cells can down-regulate the expression of sIg LCs, especially in germinal center B cells and therefore populations with negative sIg LC can be seen also in RLP [16]. Presence of dual positive sIg LCs has also been suggested as indicative of BCL [19, 23]. Currently it is still not clear if dual positive sIg LCs are only a technical problem due to unspecific staining of Fc receptors [24, 25] or real biological phenomena [19]. In order to improve cytopathological diagnostics in lymphoproliferative disorders with inconclusive sIg LC in the absence of specific lymphoma immunophenotype cytoplasmic immunoglobulin (cIg) LCs or Bcl-2 could be helpful. Moreover, molecular studies especially polymerase chain reaction (PCR) clonality analysis designed for detection of immunoglobulin heavy chain gene (*IGH*) rearrangement, may be essential for the diagnosis [26–30]. Authors have reported that high expression of intracytoplasmic Bcl-2 protein [31–33] and detection of clonality by determination of cytoplasmic κ/λ ratio [34, 35] support a lymphoma diagnosis.

In the presented study, we wanted to test if additional FCI and molecular tests performed on fine needle aspiration (FNA) material could help to distinguish between BCL and RLP whenever sIg LC determination by FCI was inconclusive. The aims of the study were: to determine which inconclusive sIg LC patterns are most often associated with BCL; to determine if any sIg LC pattern is associated with a specific histological type of BCL; to evaluate the diagnostic value of cIg LC and Bcl-2 determination using FCI; to evaluate the diagnostic value of *IGH* and T-cell receptor (TCR) gene rearrangements studies.

## Methods

The study was approved by The National Medical Ethics Committee of the Republic of Slovenia (109/02/14) and was performed in compliance with the Helsinki declaration.

Our study included 98 FNA lymph node cases suspicious for primary or secondary BCL in which determination of sIg LCs by FCI was inconclusive. The second criterion for inclusion in the study was completion of *IGH* and TCR gene rearrangement studies. Our study group represented 3.3 % of 2938 lymph node cases evaluated by FCI at the Department of Cytopathology, Institute of Oncology Ljubljana, Slovenia during the period from March 2006 to March 2012. Determination of sIg LCs was judged inconclusive when populations of B cells were observed in the negative or in the dual positive area of the κ and λ dot plot histograms or when the ratio between κ and λ LCs could not be reliably determined. The negative or dual positive populations of B cells were either solitary or present next to populations of κ and/or λ positive B cells.

In all cases included in the study gene rearrangement studies of *IGH* and TCR genes were performed by PCR. In selected cases, based on the cytopathologist’s decision, the expression of Bcl-2 and cIg LCs were determined by FCI. The flow cytometric histograms of Bcl-2 expression were evaluated by visual approach. The criterion of Bcl-2 overexpression was that B cells showed greater Bcl-2 expression than T cells. The Bcl-2 expression was categorized as not overexpressed when B cells showed a lower or similar level of Bcl-2 expression as T cells. CIg LC staining results were conclusive, when they indicated the presence of a monotypic or polytypic B cell population. B cell populations were considered monotypic when the ratio between κ and λ LCs was larger than 6:1 or lower than 1:3, and polytypic when the criteria for monotypicyty was not fulfilled [1].

From patient’s hospital records we obtained clinical and follow up data as well as cytological and histological diagnoses for all patients included in the study. Cytological diagnoses were based on morphological features of lymphoid cells and FCI results, namely on the aberrant immunophenotypes and/or on the presence of monotypic B cell populations. Final diagnoses were based either on histological results or on cytological results and follow up data. The cytological diagnosis of lymphoma was considered correct when it was in accordance with the clinical course of the disease. The case was considered RLP when lymphadenopathy resolved itself without treatment. Follow up data were registered from the date of diagnosis until the end of the study period (January 2015) or until death of the patient. Statistical analysis of results was performed using descriptive statistics.

### Flow cytometric studies

From each FNA lymph node sample two smears were prepared, one for Giemsa, the other for Papanicolaou staining. The remaining sample was used for the preparation of cell suspension. The needle and syringe were rinsed with 1 ml of cell culture medium (4.5 % bovine serum albumin, 0.45 % EDTA in phosphate buffer solution with 90,000 IE/ml of penicillin, and 4 g/ml of garamycin). The number of cells in suspension was assessed by using an improved Neubauer cell counting chamber (Brand GmbH, Wertheim, Germany). Samples for four-color FCI were prepared according to the protocol adopted for cytological samples at the Institute of Oncology, Ljubljana, Slovenia [36]. The samples were first filtered through 50 μm pore filter (CellTrics, Partec GmbH, Germany). In cases of low cellularity we used whole sample. Otherwise 200,000 cells, and 1.5 ml buffer (Cell Wash –BD Biosciences) were put in each test tube, mixed and centrifuged at 1500 rpm for 5 min (Hettich, Universal 32 centrifuge, Germany). The supernatant was discarded and appropriate amounts (3 or 5 μl) of antibodies were added as predetermined by titration. We used monoclonal antibodies against CD45, CD19, CD20, CD3, CD10, CD5, CD23, FMC7, κ and λ LCs (BD Biosciences). The samples were then mixed and incubated in darkness for 20 min. After incubation, 1.5 ml of buffer (Cell Wash – BD Biosciences) was added to the sample, mixed and centrifuged at 1500 rpm for 5 min (Hettich, Universal 32 centrifuge, Germany). The supernatant was discarded and 300 μl of buffer (Cell Wash – BD Biosciences) was added.

For the determination of Bcl-2 and cIg LCs, samples were fixed and permeabilized with Fix & Perm cell permeabilization kit, according to the manufacturer’s recommendations (Invitrogen, USA). Three-color combination of 6 or 10 μl of antibodies was used for Bcl-2 staining (Bcl-2; DAKO, Denmark, CD19, CD45; BD Biosciences,) and four-color combination of antibodies for cIg LCs (κ, λ, CD19, CD10; BD Biosciences,). We ran isotype controls (BD Biosciences) with each analysis.

The samples were acquired using four-color flow cytometer FACSCalibur (BD Biosciences) or six-color flow cytometer FACSCanto II (BD Biosciences). During sample acquisition at least 20,000 CD19 positive events were collected. For low cellularity samples all CD19 positive events were collected. The measurement results were analyzed using CellQuest (BD Biosciences) or BD FACSDiva software (BD Biosciences). For result analysis combined side scatter (SSC) and surface marker (CD19) gating was used.

### IGH and TCR gene rearrangement studies

PCR clonality analysis of lymphoid cells was used to study *IGH* and TCR gene rearrangement. Genomic DNA from FNA samples was isolated using HighPure PCR Template Preparation Kit (Roche Applied Science, Penzberg, Gremany) according to the method recommended by the manufacturer. The amount and the purity of the extracted DNA were assessed by using spectrophotometer at *A*_*260nm*_*/A*_*280nm*_ (NanoDrop, ThermoScientific, Wilmington, USA). Clonality analysis was performed using in-house and BIOMED-2 clonality assays – ABI Fluorescence Detection (Identiclone; InVivo Scribe Technologies, San Diego, CA, USA). For 32 cases analyzed before the implementation of BIOMED-2 method, in-house method was used. In this method two consensus oligonucleotide primers against VH-FR3 and JH region were used for amplifications of *IGH* rearrangements. Two primer sets including nine consensus primers were used to amplify the Vγ and Jγ gene segments of TCR-γ locus [37]. Clonality assessment was performed by using BIOMED-2 clonality assay according to the instructions of the manufacturer and as previously described [28]. The products were size-fractionated using polyacrylamide gel electrophoresis or capillary gel electrophoresis on an ABI 3500 genetic analyzer (Applied Biosystems, Foster City, CA, USA) and analyzed by fragment analysis (GeneScan).

## Results

### Clinical data and pathological findings

The 98 FNA cases included in the study were obtained from 98 patients, 61 females and 37 males. The age of the patients ranged from 4 to 94 years (average 49 years). Follow up ranged from 35 to 104 months (average 66 months). Based on the final diagnoses our study group included 30 patients with BCL and 68 patients with RLP (Table 1). In 83 % of cases cytopathological diagnosis was concordant with histology and/or follow up. There were 9 correctly suspicious diagnoses, six incorrectly suspicious, one false negative and one false positive diagnosis.

**Table 1:** Correlation between cytological and final diagnoses

	Cytological diagnoses					
Final diagnoses	DLBCL	FL	MZL	BCL-NOS	Suspicious for lymphoma	RLP
DLBCL (11)	7	-	-	-	4	-
FL (7)	-	5	-	2	-	-
MZL (10)	-	-	4^a^	1	4	1
BCL-NOS (2)	1	-	-	-	1	-
RLP (68)	-	-	-	1	6	61
TOTAL (98)	8	5	4	4	15	62

*BCL* B-cell lymphoma, *DLBCL* diffuse large B-cell lymphoma, *FL* follicular lymphoma, *MZL* marginal zone lymphoma, *NOS* not otherwise specified, *RLP* reactive lymphocytic proliferations

^a^One case composite lymphoma: marginal zone and T-cell lymphoma

Histological examination was performed in 40 patients, 25 (63 %) patients had BCL, 14 (35 %) had RLP, and one (2 %) biopsy was not representative. Histologically confirmed BCL included nine diffuse large B-cell lymphomas (DLBCL), six marginal zone lymphomas (MZL), 7 follicular lymphomas (FL), 2 B-cell lymphomas not otherwise specified, and 1 composite MZL and T-cell lymphoma. In the patient with non- representative biopsy the final diagnosis was DLBCL, confirmed by cytology and follow-up data (Table 1).

Cytological diagnoses were consistent with histology in 26/40 cases (65 %): in 15/25 lymphomas and in 11/14 RLP. There were nine correct suspicious cytological diagnoses, three incorrect suspicious and 1 false negative.

In 58 patients only cytological examination was performed. We diagnosed 50 reactive lymphocytic proliferations and five lymphomas: two MZL, 1 DLBCL, 2 B-cell lymphomas not otherwise specified. Three cases were signed out as suspicious for lymphoma. Cytological diagnoses of all 50 RLP and 4/5 lymphomas were concordant with follow up data. However, lymphoma was not confirmed in all three patients with suspicious cytological diagnosis of lymphoma and neither in one patient with positive cytological diagnosis.

### Flow cytometric findings

**Fig. 1:**
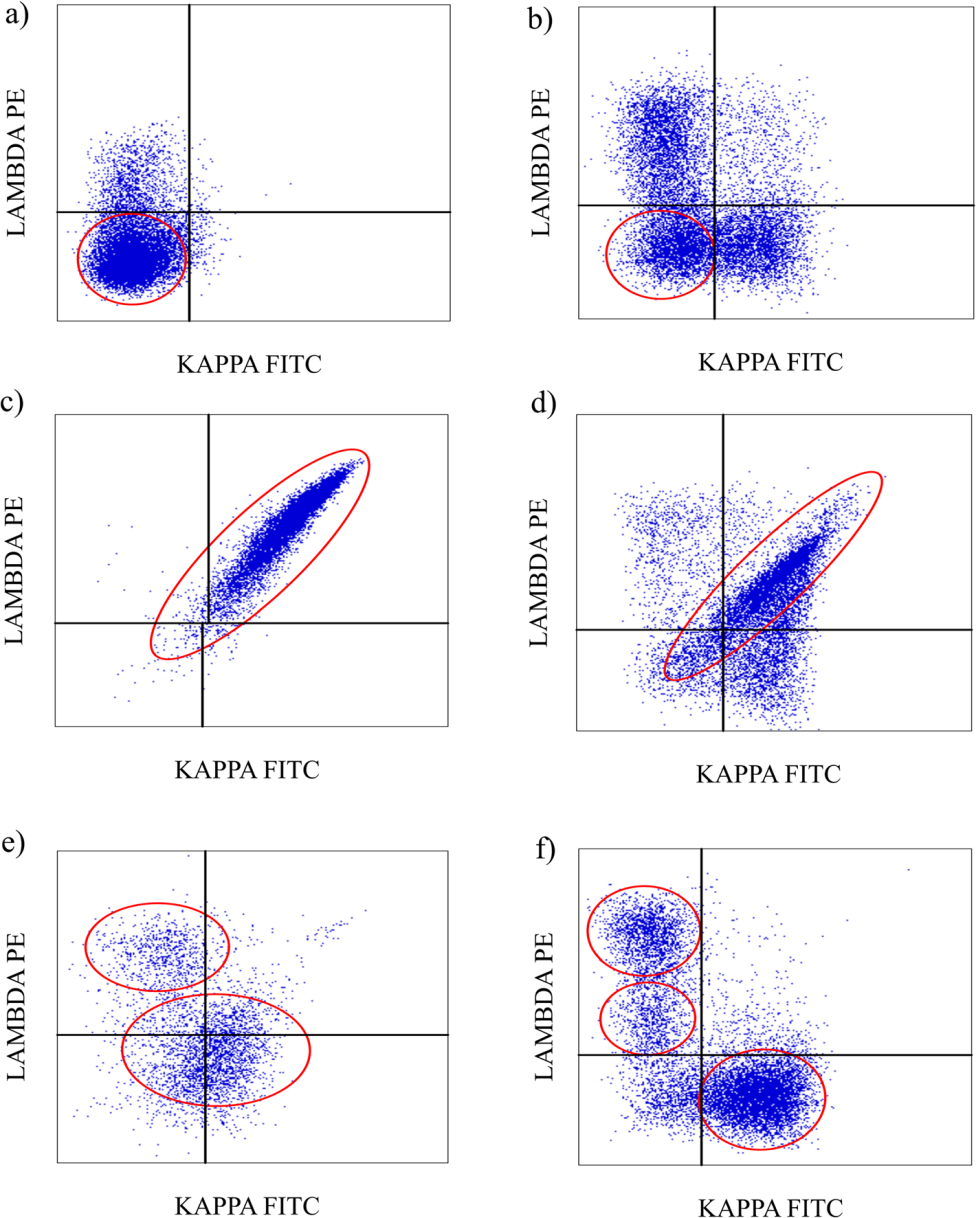
Flow cytometric dot plots showing patterns of sIg LCs. Negative (**a**,**b**), dual positive (**c**,**d**), and difficult to interpret (**e**,**f**)

According to the patterns of sIg LCs determined by FCI we divided cases into three main groups, negative (Fig. 1a, b), dual positive (Fig.1c, d), and difficult to interpret (Fig.1e, f).

SIg LCs were negative in 63/98 (64 %) cases and dual positive in 20/98 (20 %) cases. The percentages of cells with negative and dual positive sIg LCs ranged from 24 to 88 % (median = 52) and 15 to 100 % (median = 80), respectively. In all cases with negative sIg LCs we observed additional cell populations either polytypic (36/63) or monotypic (27/63) (Fig. 1a, b). Five of cases with additional monotypic populations and five of those with polytypic populations were BCL. In cases with dual positive sIg LCs 14 cases had only the population of dual positive sIg LCs (Fig. 1c) and six cases also had polytypic populations (Fig. 1d). Four/6 cases with additional polytypic populations were BCL.

In 15 (15 %) cases sIg LCs were difficult to interpret because of unusual patterns or because too few CD19 positive cells were acquired.

**Fig. 2:**
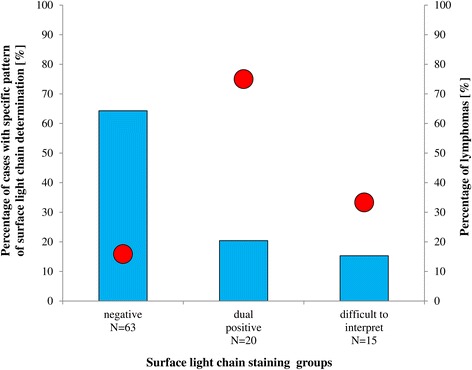
Percentage of lymphomas in each group of sIg LCs. Cases with specific patterns of sIg LCs determined by FCI (blue columns, left axis) and the percentage of lymphomas within these three groups (red dots, right axis)

Figure 2 shows the percentage of BCL within the three groups. The percentage of BCL was highest in the dual positive group (75 %; 15/20) as compared to the negative (16 %; 10/63), and the difficult to interpret group (33 %; 5/15).

DLBCL, FL and MZL were found among all the inconclusive patterns of sIg LCs without a clear predominance of any lymphoma type within a particular group (Table 2). Five/7 FL were of high grade, two in the negative and three in the dual positive group.

**Table 2:** Types of lymphomas detected in negative, dual positive and difficult to interpret groups

Type of lymphoma	Negative	Dual positive	Difficult to interpret	Total
DLBCL	3	5	3	11
FL	3	3	1	7
MZL	3	6^a^	1	10
BCL, NOS	1	1	0	2
Total	10	15	5	30

*BCL* B-cell lymphoma, *DLBCL* diffuse large B-cell lymphoma, *FL* follicular lymphoma, *MZL* marginal zone lymphoma, *NOS* not otherwise specified

^a^One case composite lymphoma: MZL and T-cell lymphoma

Results of cIg LC determination were available in 29/98 cases with inconclusive sIg LCs: for 19 (30 %) cases in the negative group, for 10 (50 %) cases in the dual positive group and none in the difficult to interpret group. Eighteen cases from the negative sIg LC group were negative also for cIg LCs and one was polytypic (Table 3). Follow-up data showed that all these cases were from RLP. However, in the dual positive sIg LC group cIg LCs were monotypic in 8/10 (80 %) cases. Seven/8 monotypic cases were from lymphomas and one case was from a patient with spontaneous regression of lymph nodes. Of the remaining two cases from the dual positive sIg LC pattern one had dual cIg LCs while the second had negative cIg LCs (Table 3). The case with dual positive LCs was lymphoma and the case with negative LCs was RLP.

**Table 3:** CIg LCs and Bcl-2 expression in groups with negative and dual positive sIg LCs patterns

			sIg LCs		
cIg LCs			Negative (*n* = 19)	Dual positive (*n* = 10)	Total (*n* = 29)
	Monotypic		0	8	8
	Polytypic		1	0	1
	Negative		18	1	19
	Dual positive		0	1	1
Bcl-2		Diagnosis	Negative (*n* = 25)	Dual positive (*n* = 9)	Total (*n* = 34)
	Overexpressed	BCL	2^a^	6^b^	8
	Not overexpressed	BCL	4	1	5
		RLP	19	2	21

*BCL* B-cell lymphomas, *RLP* reactive lymphocytic proliferations, *sIg LCs* surface immunoglobulin light chains, *cIg LC* cytoplasmic immunoglobulin light chains; ^a^2 FL; ^b^2 DLBCL, 2 FL, 2 MZL

Bcl-2 results were available for 34/98 cases of our study group, 13/30 cases of lymphomas and 21/68 cases of RLP. Bcl-2 overexpression was present in 8/13 lymphomas. None of the RLP showed Bcl-2 overexpression. Expression of Bcl-2 in lymphomas and reactive lymph nodes within different sIg LC patterns is shown in Table 3.

### IGH gene and TCR gene rearrangement studies

**Fig. 3:**
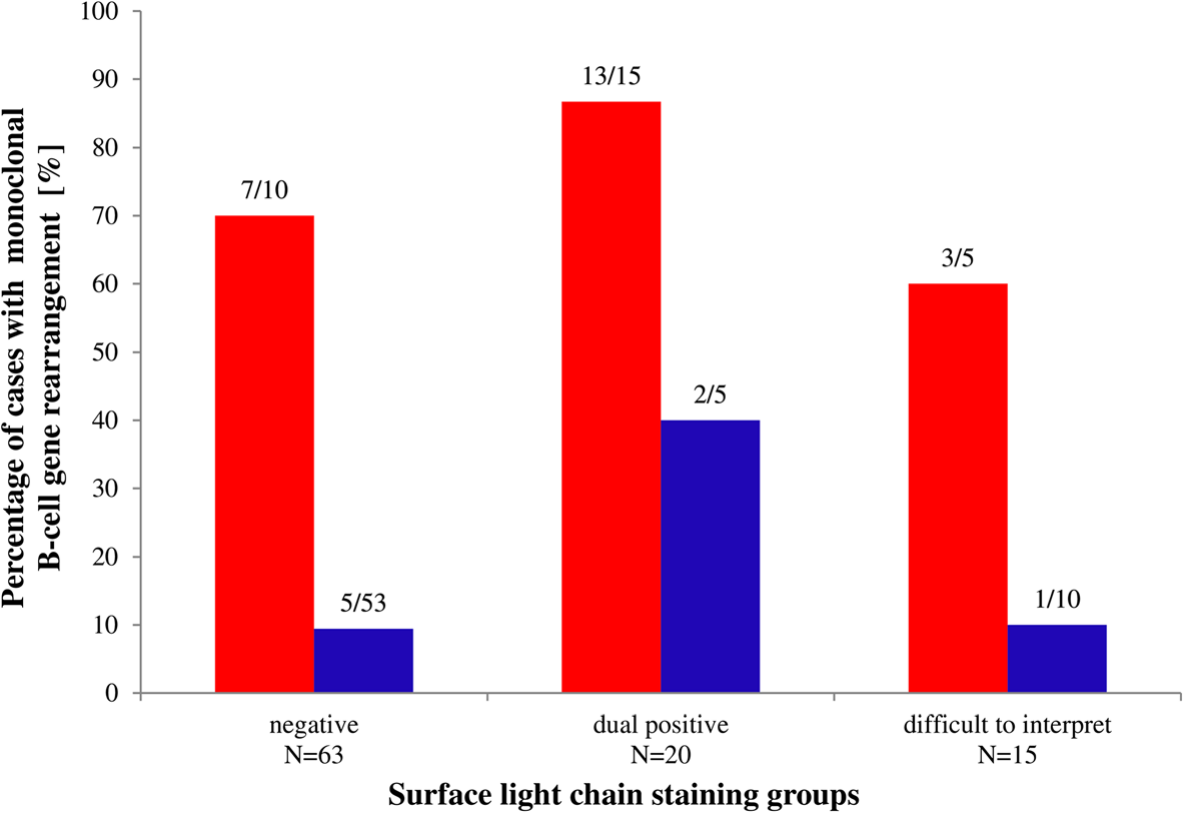
Results of IGH gene rearrangement studies in three different sIg LC patterns. The red columns represent the proportion of cases with monoclonal B cells in BCL. The blue columns represent the proportion of cases with monoclonal B cells in RLP. Final diagnoses are considered

IGH gene rearrangement results were available for all 98 cases of our study. 32/98 (33 %) cases were monoclonal and 66/98 (67 %) were polyclonal. Monoclonal B cells were found in 23/30 (77 %) cases with final diagnosis of BCL and in 8/68 (12 %) RLP. In addition, TCR gene rearrangement studies showed that 12/98 (12 %) cases had also monoclonal T-cell populations in both BCL (3/30; 10 %) and in RLP (9/68; 13 %) (Table 4). One of lymphomas with monoclonal T cells was composite MZL and T-cell lymphoma. The results of *IGH* and TCR gene rearrangement studies for cases in negative, dual positive and difficult to interpret sIg LC staining groups are presented in Table 4. Differences in the percentages of lymphoma and RLP in which IGH gene rearrangement studies detected monoclonal B cell populations are shown in Fig. 3.

**Table 4:** Monoclonal gene rearrangement results in negative, dual positive, and difficult to interpret surface light chains staining group

		sIg LCs staining groups			
Gene rearrangement	Final diagnosis	Negative (N_1_/N)	Dual positive (N_1_/N)	Difficult to interpret (N_1_/N)	Total (N_1_/N)
monoclonal B cells	B-cell lymphoma	7/10	13/15	3/5	23/30
	Reactive	5/53	2/5	1/10	8/68
	total	12/63	15/20	4/15	30/98
monoclonal T cells	B-cell lymphoma	1/10	2/15^a^	0/5	3/30
	reactive	9/53	0/5	0/10	9/68
	total	10/63	2/20	0/15	12/98

*N*_*1*_ lymphoma cases, *N* all cases in the group, *sIg LCs* surface immunoglobulin light chains, *BCL* B-cell lymphomas, *RLP* reactive lymphocytic proliferations

^a^Case of composite MZL and T-cell lymphoma

## Discussion

This study showed that additional FCI tests performed on FNA samples from lymph nodes helped to distinguish between BCL and RLP in cases with inconclusive sIg LC determination by FCI. Morphology coupled with cIg LC determination and/or Bcl-2 expression was able to give a correct diagnosis in 83 % of cases. Molecular tests, which were not included in the decision on the original cytological diagnoses, could have contributed positively in 85 % and would have been misleading in 15 % of cases.

### Accuracy of cytopathologic diagnoses

The results of our study are similar to those reported by Laane et al.[33]. They were able to correctly classify 89 % of their cases which is slightly better than our result. However, their study was not selective and did not include only problematic cases with inconclusive sIg LCs. In addition to the 83 % correct diagnoses. The one case which we labeled as the false positive cytological result was morphologically a lymphoma which regressed completely without therapy. In addition to the small B cell population with dual sIg LCs there was a monotypic population of large B cells with positive surface and cytoplasmic κ LCs, expressing CD5 and lacking CD10. It is doubtful whether this was a true false positive result because spontaneous regression of non-Hodgkin lymphoma has been reported previously. Krikorian et al.[38] have reported seven cases of lymphoma with favorable histologic subtypes which regressed without treatment. Iwatani et al. [39] reported a case of an aggressive high grade lymphoma of the breast which regressed after core biopsy.

### Patterns of inconclusive sIg LCs

Our FCI findings showed three main groups of inconclusive sIg LCs patterns in lymphomas and in RLP (Fig. 1). No specific lymphoma type was associated with a particular sIg LC pattern. The dual positive sIg LC pattern showed the highest risk for BCL, since 75 % of cases in this group were lymphomas. The presence of dual positive sIg LCs has been previously reported as case reports, mostly in chronic lymphatic leukemia (CLL) [18, 20, 22, 23] as well as individual cases of MALT lymphomas [40, 41]. Only one report describes an aggressive large cell lymphoma, histologically undetermined [19]. In contrast to the majority of indolent lymphomas reported in the literature our group of 15 lymphomas with dual sIg LCs included 5 aggressive DLBCL. It has been suggested that dual positive sIg LC pattern may be the result of unspecific staining which could be minimized by more extensive washing of the sample or by prolonged incubation in isotonic buffer [24]. However, according to our experience such procedures result in some cell loss which may hinder FCI determinations in samples which are not highly cellular.

Only 16 % of cases in the negative sIg LC group of our study were BCL. Therefore we concluded that this pattern of LC expression has a low risk for lymphoma. These results are in contrast with the results of some authors who claim that negative sIg LCs can be used as a substitute of monotypicyty because such cases are found mostly among lymphomas [12, 13]. Zhao [16] warned that benign follicular hyperplasia can also have negative sIg LC pattern. However, he found only three such cases among 101 reactive lymphadenopathies. Furthermore, frequency of lymphomas with negative sIg LCs reported in the literature depends on the criteria used and ranged from 3.4 to 12.2 % as stated by Li et al.[13]. His criterion was complete lack of sIg LCs and the frequency of such lymphomas was 2.3 %. In the study by deMartini et al. [17] the criterion was less than 15 % κ positive and less than 10 % λ positive cells and therefore the percentage of sIg LC negative lymphomas was 13 %. One of the reasons for low percentage of lymphomas with negative sIg LCs in our study is absence of CLL and mantle cell lymphoma. In our daily practice we did encounter occasional cases of these two lymphomas which lacked sIg LCs. However, such cases expressed a typical immunophenotypes for CLL and therefore additional FCI or molecular investigations were not performed.

We have found no reports on lymphadenopathies expressing sIg LCs that are difficult to interpret. It seems that such cases are usually excluded from studies [13]. Since the majority of such cases in our study had low cellularity we were not able to do additional FCI analysis.

### Determination of cIg LCs

Investigation of cIg LCs is also not reported frequently. We have found only one report which described cIg LCs in a series of lymphomas [34]. Lewis et al. [34] studied 30 cases of CLL because week expression of sIg LCs is one of the five criteria in the international scoring system to help discriminate between CLL and other BCL. They found that 6/7 cases with negative sIg LCs had positive cIg LCs. This result has been commented by Smith et al. [14] in their report on a case of FL which lacked both sIg and cIg LCs. Smith et al. [14] speculated that the results of Lewis et al. [34] suggested that investigating both types of light chains would significantly increase the chance of detecting LC restriction. Since the study only included CLL it was not clear whether such conclusion was applicable to other types of lymphomas. Even though we were able to investigate the presence of cIg LCs only in 30 % of cases, our results do shed some light on the usefulness of such testing. We were able to detect cIg LC restriction in 8/9 lymphomas where sIg LCs had dual positivity. In the group of cases with negative sIg LCs 18/19 cases also lacked cIg LCs and one case was polyclonal. All these 19 cases were from RLP. Unfortunately our study does not include any lymphoma with negative sIg LCs in which cIg LCs were determined. However, we have a few lymphoma cases in our files with negative sIg LCs and negative cIg LCs (unpublished data). Absence of sIg and cIg LCs is rare in lymphomas. In addition to CLL and FLs mentioned above, the lack of cIg LCs has also been reported in two Burkitt lymphomas [42].

### Bcl-2 expression

Bcl-2 expression is mainly used as an ancillary method in the differentiation between FL and benign follicular hyperplasia. The presence of overexpression of Bcl-2 in other lymphoma types is not well documented. Our results are concordant with data from the literature [31, 32] that Bcl-2 is overexpressed in approximately 85 % of FL and not overexpressed in reactive lymph nodes (Table 3). However, Laane et al. [33] found two cases among 137 reactive hyperplasias in which a subpopulation of B cells expressed high levels of Bcl-2. The study was performed on FNA material and these two cases were signed out as suspicious for lymphoma. Our study of Bcl-2 expression included eight cases of lymphoma other than FL and overexpression was observed in half of them (Table 3). The number of individual lymphoma types investigated for Bcl-2 overexpression in our study and in the report of Cook et al. [31] is too low to draw any important conclusions. Laane et al. [33] on the other hand, investigated 117 non-FL for Bcl-2 expression. They found high levels of Bcl-2 expression in all 88 indolent lymphomas, however, these expressions were lower than in FLs. Among the 29 aggressive lymphomas of their study, high levels of Bcl-2 expression were present only in nine cases which were CD10 positive.

The main weakness of our study was the fact that cIg LC determination and Bcl-2 expression were performed only in 30 and 35 % of cases respectively. Furthermore, presence of cIg LCs was not performed in any lymphomas with negative sIg LCs. The reason for this drawback is the criterion for inclusion of cases in our study which required that all cases with inconclusive sIg LC also have results of *IGH* and TCR gene rearrangement studies. In this way we lost some cases with inconclusive sIg LCs in which cIg LCs and/or Bcl-2 expression were performed. This is also the reason for the absence of CLL and mantle cell lymphomas from our study as well as the absence of lymphomas with negative sIg and cIg LCs.

### Molecular tests

The outcome of our molecular tests was somewhat disappointing since they were concordant with final diagnoses in 85 % and misleading in 15 % of our cases. They showed monoclonality in only 77 % of BCL. We were unable to detect monoclonality in 3 DLBCL, 3 FL, and 1 MZL. On the other hand, monoclonality of B cells was present also in 12 % of RLP, while monoclonal populations of T cells were present in 12 % of all our cases.

With the use of molecular test our detection rate of BCL was lower than reported by EuroClonality/BIOMED-2 group where clonal IGH-gene rearrangement was found in 91 % of BCL [29]. The frequency of monoclonal B cell populations in RLP was similar to the reported 10 % of the EuroClonality/BIOMED-2 group. There are several reasons for under-detection of BCL by the PCR technique such as suboptimal sampling, few lymphoma cells in reactive background, the use of imperfect consensus primers, atypical rearrangement or somatic hypermutation [43, 44]. The somatic hypermutation is most common among malignant lymphomas that arise from B cells exposed to antigen, such as FL and DLBCL or MZL [44] and was most probably the main cause for under-detection of monoclonality in our study. The second reason was probably the use of less sensitive in-house PCR clonality analysis in one third of our cases. To enhance the sensitivity of PCR clonality analysis of B cells, immunoglobulin kappa light chain gene rearrangement studies are advisable [45].

PCR based *IGH* and TCR gene rearrangement studies are not frequently used in routine cytological diagnostics of BCL because FCI has been proven as a sensitive and specific ancillary technic for differentiation between BCL and RLP [1, 46]. However, FCI may not be helpful in cases of low cellularity, whenever there are few lymphoma cells in a reactive background or when FCI results are inconclusive. Safley et al. [47] therefore proposed a combined diagnostic algorithm including microscopic analysis, FCI and molecular evaluation for the diagnosis of BCL by FNA. According to this algorithm PCR clonality analysis of lymphoid cells and detection of specific translocations by FISH should be reserved only for cases in which FCI fails to detect a monotypic B cell population [47]. Ribera et al. [48] have recently used a similar approach in 102/722 (14 %) cases of lymphoproliferative disorders with inconclusive FCI results. In their series PCR based *IGH* clonality analysis implied malignancy, because all monoclonal cases were BCL. However, 26.3 % (5/19) of BCL were missed because PCR results were polyclonal, 26.3 % (5/19) were missed because PCR result were inconclusive or not informative. Despite of that the authors concluded that PCR clonality analysis of lymphoid cells could be helpful if the results are interpreted along with clinical and morphological findings [48]. Our results on PCR based *IGH* and TCR gene rearrangement studies are in concordance with the results published by Safley et al. [47] and Ribera et al. [48].

## Conclusion

In our study determination of cIg LCs, Bcl-2 expression and PCR clonality analysis improved accuracy of cytological diagnoses in BCL with inconclusive sIg LCs. Dual positive sIg LCs were more frequent in BCL than in RLP and determination of cIg LCs showed monotypic populations in most cases. Therefore, we propose that cIg LC should be determined first in such cases since determination of Bcl-2 expression might not be necessary. On the other hand, our results showed that the majority of RLP and lymphomas with negative sIg LCs also had negative cIg LCs . Therefore, we propose that Bcl-2 expression should be a priority in such cases. However, lack of cIg LCs and lack of Bcl-2 overexpression do not rule out lymphoma. When cIg LCs and Bcl-2 are not helpful PCR clonality analysis should be performed. Monoclonal result implies lymphoma but is not an absolute proof and polyclonal result does not rule out lymphoma.
